# Exosomes derived from M0, M1 and M2 macrophages exert distinct influences on the proliferation and differentiation of mesenchymal stem cells

**DOI:** 10.7717/peerj.8970

**Published:** 2020-04-24

**Authors:** Yu Xia, Xiao-Tao He, Xin-Yue Xu, Bei-Min Tian, Ying An, Fa-Ming Chen

**Affiliations:** State Key Laboratory of Military Stomatology, National Clinical Research Center for Oral Diseases, Shaanxi Engineering Research Center for Dental Materials and Advanced Manufacture, Department of Periodontology, School of Stomatology, Fourth Military Medical University, Xi’an, Shaanxi, P. R. China

**Keywords:** Macrophages, Bone marrow mesenchymal stem cells, Exosomes, Cell proliferation, Cell differentiation

## Abstract

**Background:**

Different phenotypes of macrophages (M0, M1 and M2 Mφs) have been demonstrated to play distinct roles in regulating mesenchymal stem cells in various in vitro and in vivo systems. Our previous study also found that cell-conditioned medium (CM) derived from M1 Mφs supported the proliferation and adipogenic differentiation of bone marrow mesenchymal stem cells (BMMSCs), whereas CM derived from either M0 or M2 Mφs showed an enhanced effect on cell osteogenic differentiation. However, the underlying mechanism remains incompletely elucidated. Exosomes, as key components of Mφ-derived CM, have received increasing attention. Therefore, it is possible that exosomes may modulate the effect of Mφ-derived CM on the property of BMMSCs. This hypothesis was tested in the present study.

**Methods:**

In this study, RAW264.7 cells were induced toward M1 or M2 polarization with different cytokines, and exosomes were isolated from the unpolarized (M0) and polarized (M1 and M2) Mφs. Mouse BMMSCs were then cultured with normal complete medium or inductive medium supplemented with M0-Exos, M1-Exos or M2-Exos. Finally, the proliferation ability and the osteogenic, adipogenic and chondrogenic differentiation capacity of the BMMSCs were measured and analyzed.

**Results:**

We found that only the medium containing M1-Exos, rather than M0-Exos or M2-Exos, supported cell proliferation and osteogenic and adipogenic differentiation. This was inconsistent with CM-based incubation. In addition, all three types of exosomes had a suppressive effect on chondrogenic differentiation.

**Conclusion:**

Although our data demonstrated that exosomes and CM derived from the same phenotype of Mφs didn’t exert exactly the same cellular influences on the cocultured stem cells, it still confirmed the hypothesis that exosomes are key regulators during the modulation effect of Mφ-derived CM on BMMSC property.

## Introduction

During the past several decades, stem cell therapy has been the focus of tissue engineering and regenerative medicine (Wei et al., 2013; Thurairajah, Broadhead & Balogh, 2017). Attributed to their advantages, mesenchymal stem cells (MSCs) stand out from multiple stem cells and become the most promising choice for both autologous and allogeneic transplantation (Maxson et al., 2012; Wei et al., 2013; Poltavtseva et al., 2018). However, the application of MSCs from bench to bedside encounters many challenges, such as low cell dose, low survival rate and poor potency (Silva et al., 2018; Regmi et al., 2019). Considerable efforts have been made to improve the regenerative efficiency of MSCs in vivo. Currently, the significance of macrophages (Mφs) in the recruitment and modulation of MSCs is well recognized (Zhang et al., 2017a; Ma et al., 2018; Cai et al., 2018).

Macrophages, essential components of innate immunity, play important roles in tissue regeneration (Mosser & Edwards, 2008; Krzyszczyk et al., 2018). In response to various stimuli, Mφs can switch phenotype from an unpolarized (M0) to a polarized (M1 and M2) state (Mantovani et al., 2013) and play unique roles in different stages of tissue healing (Oishi & Manabe, 2018; Pajarinen et al., 2019). Generally, M1 Mφs contribute to the debridement of wounds and exert pro-inflammatory functions. In contrast, M2 Mφs exert anti-inflammatory functions and facilitate tissue repair (Murray et al., 2014; Shapouri-Moghaddam et al., 2018). During the past few years, accumulative studies have confirmed the modulating effect of Mφs on MSCs (Yu et al., 2016; Grotenhuis et al., 2016). Our previous study also revealed that cell-conditioned medium (CM) generated by differently polarized Mφs exerted different influences on BMMSC cellular behaviors in vitro (He et al., 2018). However, the exact mechanism remains unclear. The dominant opinion is that cytokines are the main contributors to Mφ function (Champagne et al., 2002; Zhang et al., 2017b); however, Ekström et al. (2013) found a modulating effect of LPS-stimulated monocyte-derived exosomes on MSCs. Therefore, to better illustrate the mechanism of Mφ-MSC cross-talk, Mφ-derived exosomes should be considered.

Exosomes are special endosomal-derived membranous microvesicles with a diameter of 50–150 nm. They are crucial mediators in intercellular communication and participate in many biological activities (Zhang et al., 2019). Exosomes, which carry proteins, lipids, nucleic acids and other cargos, can be released into the extracellular milieu and internalized by target cells, in which they modulate cellular behaviors (Jan et al., 2019). The biocompatibility, stability and capacity to transport bioactive components and overcome biological barriers indicate the great potential of exosomes as suitable therapeutic agents (Liu & Su, 2019). For example, it was found that maturing osteoclast-derived exosomes transport RANK which can bind osteoblastic RANKL and promote bone formation (Ikebuchi et al., 2018). Actually, increasing evidence has suggested that Mφ-derived exosomes are important regulators in many physiological processes (McDonald et al., 2014; Saha et al., 2016; Wei et al., 2019). However, the role of different phenotypes of Mφ-derived exosomes in the regulation of MSC functions remains ambiguous.

Based on these previous studies, we hypothesized that exosomes are key regulators during the modulation effect of Mφ-derived CM on bone marrow mesenchymal stem cells (BMMSCs). Our study also aims to further clarify the function of exosomes derived from different phenotypes of Mφs (M0-Exos, M1-Exos and M2-Exos) on the proliferation and differentiation of BMMSCs. The outcomes are expected to improve our understanding of Mφ-MSC cross-talk and contribute to better modulation of MSC potency in tissue regeneration.

## Materials and Methods

### Isolation and culture of mouse BMMSCs

Male C57BL/6 mice (6–8 weeks) were purchased from the Laboratory Animal Research Centre of the Fourth Military Medical University. Animals used in this study were approved by the Animal Use and Care Committee of the Fourth Military Medical University (IACUC-20180804). According to previously reported methods (Huang et al., 2015), the mice were sacrificed by cervical dislocation and the femurs and tibias dissected. After two washes with phosphate buffer solution (PBS), bone marrow cells were flushed from the bones into 10-cm culture dishes using alpha-minimal essential medium (α-MEM; Invitrogen, Carlsbad, CA, USA) supplemented with 20% fetal bovine serum (FBS, Hangzhou Sijiqing Biological Engineering Materials, Zhejiang, China) and 1% penicillin and streptomycin (Sigma–Aldrich, St. Louis, MO, USA). Then, the dishes were incubated at 37 °C in a 5% CO_2_ incubator. The medium was refreshed every 3 days to remove nonadherent cells. When the primary cells reached 70–90% confluence, they were digested with 0.25% trypsin (Invitrogen, Carlsbad, CA, USA) and passaged. Cells from the 2nd or 3rd passage were used in the following experiments.

### Identification of mouse BMMSCs

The isolated primary BMMSCs were subjected to flow cytometry analysis, colony-forming assay, EdU (5-ethynyl-2′-deoxyuridine) incorporation assay, cell counting kit-8 (CCK-8) assay, Alizarin red S staining, Oil red O staining and Alcian blue staining to identify the phenotype. The results were showed in the Supplemental File. The flow cytometry analysis revealed that these cells were strongly positive for MSC markers, such as CD105, Sca-1, CD73 and CD90, but were negative for hematopoiesis-related markers, CD34 and CD45 (Fig. S1A). Toluidine blue staining revealed that these cells possessed the ability to form new colony units (Fig. S1B). Furthermore, these cells exhibited decent proliferative potential, as evidenced by the results of the EdU and CCK-8 assays (Figs. S1C and S1D). Alizarin red S staining showed that mineralized nodules formed after osteogenic induction (Fig. S1E). Oil red O staining showed the formation of lipid-rich vacuoles after adipogenic induction (Fig. S1F), and Alcian blue staining showed acidic proteoglycan formation after chondrogenic induction (Fig. S1G). All of these observations confirmed the multipotent differentiation ability of the BMMSCs.

### Culture of the Mφs and Mφ polarization

The mouse Mφ cell line RAW264.7 (ATCC Cat# TIB-71, RRID: CVCL_0493) was used in the present study and cultured in α-MEM supplemented with 10% FBS. For each sample, 2 × 10^6^ cells were seeded into a 10-cm culture dish. As previously reported (He et al., 2018), lipopolysaccharide (LPS) at a concentration of 200 ng/ml plus interferon-gamma (IFN-γ) at a concentration of 10 ng/ml was used to induce Mφ polarization into the M1 phenotype, while IL-4 at a concentration of 20 ng/ml was used to induce Mφs toward the M2 polarization. All cytokines were purchased from PeproTech, Princeton, NJ, USA. As a control, RAW264.7 cells incubated in medium supplemented with PBS were considered M0 Mφs. Following a 24-h induction, the phenotypes of polarized cells (as stimulated by LPS plus IFN-γ or IL-4) and the unpolarized cells (PBS) were identified by flow cytometry analysis, quantitative real-time polymerase chain reaction (qRT-PCR) and enzyme-linked immunosorbent assay (ELISA).

### Isolation and characterization of M0, M1 and M2 Mφ-derived exosomes

To isolate exosomes from the M0, M1 and M2 Mφs, exosome-depleted FBS was generated by ultracentrifugation of FBS at 100,000×*g* for 70 min with an L-80 ultracentrifuge (45 Ti rotor) from Beckman Coulter (Brea, CA, USA) (Hoshino et al., 2015), which removed the bovine exosomes from the FBS. Although we do not have nanoparticle tracking data of FBS before and after ultracentrifugation, we trust that FBS was effectively depleted of exosomes since other studies used similar methods (Kobayashi et al., 2014; Lee et al., 2019). Following a 24-h incubation with or without M1/M2 induction, the culture medium was discarded, and the cells were washed twice with PBS to remove remaining cytokines. Then, α-MEM supplemented with 10% exosome-depleted FBS was used to further culture the cells. After 24 h, the CM generated by the M0, M1 or M2 Mφs (termed CM0, CM1 and CM2) was collected separately. Each CM sample was first centrifuged at 2,000×*g* (30 min at 4 °C) to remove cells and debris. After the CM was transferred into a new tube, 0.5 volume of the total exosome isolation (from cell culture medium) reagent (Invitrogen, Carlsbad, CA, USA) was added to each CM supernatant. Then, the CM/reagent mixtures (CM0, CM1 and CM2) were vortexed and incubated at 4 °C overnight as described in the instructions. Finally, each mixture was centrifuged at 10,000×*g* for 60 min at 4 °C. Following the removal of the supernatant, the exosomes at the bottom of each tube (M0-Exos, M1-Exos or M2-Exos) were resuspended with PBS (exosomes isolated from 1 ml of CM of each sample were suspended in 100 μl of PBS).

#### Transmission electron microscopy

Freshly isolated exosomes were dropped on special copper grids, where they dried at room temperature. Then, the samples were subjected to negative staining with 1% aqueous uranyl acetate for 5 min and washed twice with deionized water. The grids were dried at room temperature before TEM analysis. The samples were visualized with a JEM-1400Plus transmission electron microscope from JEOL (Tokyo, Japan).

#### Nanoparticle tracking analysis

The M0-Exos, M1-Exos and M2-Exos were sent to a company (Wuhan GeneCreate Biological Engineering Co., Ltd., Wuhan, China) for nanoparticle tracking analysis. In brief, the exosome samples were diluted to an optimal concentration, and the size and number were determined with a NanoSight NS 300 system (NanoSight Technology, Malvern, UK).

#### Western blotting analysis

Western blot assays were performed to measure the exosome surface markers CD9, CD63, CD81 and Alix on M0-Exos, M1-Exos and M2-Exos. The exact procedures of the Western blot assay were previously described (Xu et al., 2019). The primary antibodies used in this study were anti-mouse CD63 antibody (Abcam, Cambridge, UK), anti-mouse CD81 antibody (Cell Signaling Technology, Danvers, MA, USA), anti-mouse CD9 antibody (Abcam, Cambridge, UK) and anti-mouse Alix antibody (Cell Signaling Technology, Danvers, MA, USA). Horseradish peroxidase (HRP)-conjugated goat anti-rabbit and goat anti-mouse (Cell CWBIO) secondary antibodies were used.

### Internalization of the exosomes by BMMSCs

PKH67 fluorescent cell linker kits (Sigma–Aldrich, St. Louis, MI, USA) were used to label the exosomes according to the manufacturer’s instructions. Then, the mixture of the exosomes and PKH67 dye was subjected to exosome spin columns (MW3000) (Invitrogen, Carlsbad, CA, USA) to remove excess dye. Finally, the PKH67-labeled exosomes were incubated with BMMSCs at 37 °C for 4 h. A confocal laser microscope (FV1000; Olympus, Tokyo, Japan) was used to observe the uptake of the exosomes by the BMMSCs.

### Cell treatment and grouping

To investigate the effects of M0, M1 and M2 Mφ-derived exosomes on the proliferation and differentiation of BMMSCs, the M0-Exos, M1-Exos or M2-Exos at a concentration of 100 μl/ml were supplemented into the complete medium or inductive medium used to culture BMMSCs, respectively. For each group, the culture medium was refreshed every other day and exosomes were added at the same time. The culture medium supplemented with the same volume of PBS were used as blank control.

### Effects of Mφ-derived exosomes on BMMSC proliferation

The proliferation ability of the BMMSCs cultured in different complete medium (Supplemented with M0-Exos, M1-Exos, M2-Exos or PBS) was determined on the basis of cell colony-forming, CCK-8 and EdU incorporation assays.

### Effects of Mφ-derived exosomes on BMMSC osteogenic differentiation

Osteogenic medium was generated by complete medium supplemented with 50 μg/ml vitamin C, 10 nM dexamethasone and 10 mM β-glycerophosphate (all purchased from Sigma–Aldrich, St. Louis, MO, USA). To assess the osteogenic potency of the BMMSCs, the cells were seeded in 12-well culture plates at a density of 2 × 10^5^ cells/well. When the cells reached 80–90% confluence, the culture medium was replaced with different osteogenic medium (Supplemented with M0-Exos, M1-Exos, M2-Exos or PBS). After 7 days of osteogenic induction, the CM of each group was collected to measure the ALP activity with an alkaline phosphatase assay kit (Jiancheng Bioengineering, Nanjing, China). The cells were fixed with 4% paraformaldehyde for 30 min and stained with a BCIP/NBT alkaline phosphatase color development kit (Beyotime Institute of Biotechnology, Haimen, China) as described in the instructions. The expression level of osteogenesis-related genes (*ALP*, *BMP-2*, *COL-1*, *OCN* and *Runx2*) were assessed by qRT-PCR analysis. In addition, the Alizarin red S staining were also conducted after induction for 14 days.

### Effects of Mφ-derived exosomes on BMMSC adipogenic differentiation

The adipogenic induction medium was generated by complete medium supplemented with 0.5 mM 3-isobutyl-1-methylxanthine, 1 μM dexamethasone, 0.1 mM indomethacin and 10 μg/ml insulin. To detect the adipogenesis ability of the BMMSCs, the cells were seeded in 12-well culture plates at a density of 2 × 10^5^ cells/well. When the cells reached 80–90% confluence, the culture medium was replaced with different adipogenic induction medium. After induction for 7 days, the adipogenic differentiation of the BMMSCs were determined with Oil red O staining and qRT-PCR assay.

### Effects of Mφ-derived exosomes on BMMSC chondrogenic differentiation

To assess the chondrogenic differentiation ability of the BMMSCs in response to various exosomes, the cells were seeded in 12-well culture plates at a density of 2 × 10^5^ cells/well. When the cells reached 80–90% confluence, the complete medium was replaced with chondrogenic differentiation medium (Cyagen Biosciences, Inc., Guangzhou, China) supplement with M0-Exos, M1-Exos, M2-Exos or PBS. After chondrogenic induction for 7 days, the chondrogenic differentiation ability were analyzed by Alcian blue staining and qRT-PCR assay.

### Flow cytometry analysis

The cell surface markers of the BMMSCs and Mφs were analyzed by flow cytometry. Briefly, the cells were trypsinized and washed with PBS. To block Fc receptors, the cells were incubated with 2% anti-mouse CD16/32 (BioLegend, San Diego, CA, USA) on ice for 10 min. Then, the cells were washed twice with PBS and incubated with specific antibodies for 30 min at 4 °C in the dark. Excess antibody was removed by washing the cells with PBS. Untreated cells were used as blank controls. The samples were then analyzed with a Beckman Coulter Epics XL cytometer (Beckman Coulter, Fullerton, CA, USA). For the characterization of BMMSCs, the following antibodies were used: PE anti-mouse Ly-6A/E (Sca-1), PE anti-mouse CD90.2, PE-anti-mouse CD73, PE anti-mouse CD105, PE anti-mouse CD34 and FITC anti-mouse CD45 (all from BioLegend, San Diego, CA, USA). FITC anti-mouse CD86 and PE anti-mouse CD206 (both from BioLegend, San Diego, CA, USA) were used for the identification of Mφs. In this experiment, pulse width measurements were used to eliminate the possibility that the detected cells were doublets of RAW 267 cells. 7-aminoactinomycin D (7-AAD), as well as isotype controls were used to exclude dead cells and the nonspecific binding of the monoclonal antibodies. Each group with no less than 10^6^ cells was gated for flow cytometric analysis.

### Quantitative real-time polymerase chain reaction

To measure the mRNA expression levels, qRT-PCR was conducted. Total RNA from the cultured cells was extracted with TRIzol reagent (Invitrogen, Carlsbad, CA, USA). According to the manufacturer’s instructions, cDNA was synthesized using PrimeScript™ RT Master Mix (Perfect Real-Time; TaKaRa, Tokyo, Japan). Then, qRT-PCR was performed using SYBR^®^ Premix Ex Taq™ II (Tli RNaseH Plus; TaKaRa, Tokyo, Japan). The primers used are listed in Table 1. The β*-actin* housekeeping gene was used to normalize the expression level of the related genes.

**Table 1:** Primer sequences for quantitative real-time polymerase chain reaction (qRT-PCR) analysis.

Primer	Full name		Sequence (5′–3′)
*IL-1*β	*Interleukin 1-*β	Forward	AAGGAGAACCAAGCAACGACAAAA
		Reverse	TGGGGAACTCTGCAGACTCAAACT
*iNOS*	*Inducible nitric oxide synthase*	Forward	CAAGCTGAACTTGAGCGAGGA
		Reverse	TTTACTCAGTGCCAGAAGCTGGA
*TNF-*α	*Tumor necrosis factor-*α	Forward	TATGGCCCAGACCCTCACA
		Reverse	GGAGTAGACAAGGTACAACCCATC
*Arg-1*	*Arginine-1*	Forward	AGCTCTGGGAATCTGCATGG
		Reverse	ATGTACACGATGTCTTTGGCAGATA
*CD206*	*CD206*	Forward	AGCTTCATCTTCGGGCCTTTG
		Reverse	GGTGACCACTCCTGCTGCTTTAG
*IL-10*	*Interleukin 10*	Forward	GCCAGAGCCACATGCTCCTA
		Reverse	GATAAGGCTTGGCAACCCAAGTAA
*ALP*	*Alkaline phosphatase*	Forward	CTTCTTGCTGGTGGAAGGA
		Reverse	AAAACGTGGGAATGATCAGC
*BMP-2*	*Bone morphogenetic protein 2*	Forward	TGACTGGATCGTGGCACCTC
		Reverse	CAGAGTCTGCACTATGGCATGGTTA
*COL-1*	*Collagen-1*	Forward	GCTGGAGTTTCCGTGCCT
		Reverse	GACCTCGGGGACCCATTG
*Runx2*	*Runt-related transcription factor-2*	Forward	AGGGAATAGAGGGGATGCATTAG
		Reverse	AAGGGAGGACAGAGGGAAACA
*OCN*	*Osteocalcin*	Forward	CTGACAAAGCCTTCATGTCCAA
		Reverse	GCGCCGGAGTCTGTTCACTA
*Adiponectin*	*Adiponectin*	Forward	TTCTGTCTGTACGATTGTCAGTGGA
		Reverse	GGCATGACTGGGCAGGATTA
*PPAR-*γ	*Peroxisome proliferator activated receptor-*γ2	Forward	TCAGGTTTGGGCGGATG
		Reverse	CAGCGGGAAGGACTTTATGTATG
*Col-2a1*	*Collagen type II* α1	Forward	CTGACCTGACCTGATGATACC
		Reverse	CACCAGATAGTTCCTGTCTCC
*Cdh2*	*Cadherin 2*	Forward	CCGTGAATGGGCAGATCACT
		Reverse	TAGGCGGGATTCCATTGTCA
*Sox9*	*SRY (sex determining region Y)-box 9*	Forward	TACGACTGGACGCTGGTGCC
		Reverse	CCGTTCTTCACCGACTTCCTCC
β*-actin*	β*-actin*	Forward	CTCTTTTCCAGCCTTCCTTCTT
		Reverse	GAGGTCTTTACGGATGTCAACG

### Enzyme-linked immunosorbent assay

After induction for 24 h, the polarized cells (M1 and M2 Mφs) were washed with PBS to remove remaining cytokines and then were cultured with fresh medium for another 24 h. Then, the culture media of M0, M1 and M2 Mφs were collected and centrifuged to remove the cells and debris. The concentrations of two different cytokines (TNFα and IL-10) secreted into the CM were then detected with ELISA kits (Neobioscience, Guangzhou, China) according to the manufacturer’s instructions.

### Colony-forming assay

Briefly, 800 cells were seeded in a 60-mM culture dish. After 14 days, the cells were washed with PBS and fixed for 30 min in 4% paraformaldehyde. Then, the cells were stained with 1% toluidine blue for 20 min at room temperature. After the cells were washed three times with PBS, photos were taken of these dishes, and the colony-forming units (CFUs) were counted under a microscope. Each CFU with ≥50 cells was quantified for statistical analysis.

### EdU (5-ethynyl-2′-deoxyuridine) incorporation assay

Bone marrow mesenchymal stem cells were seeded in a 12-well culture plate at a density of 2 × 10^5^ cells per well. When the cells reached 70–80% confluence, the EdU incorporation assay was performed according to the manufacturer’s protocol with a BeyoClick™ EdU Cell Proliferation Kit with Alexa Fluor 594 (Beyotime Institute of Biotechnology, Haimen, China). The cells were visualized with a LeicaTCS SP5 X confocal microscope (Leica, Germany) and photographed.

### Cell counting kit-8 assay

Bone marrow mesenchymal stem cells were seeded in a 96-well culture plate at a density of 3,000 cells per well, and the culture medium was refreshed every other day. Every day at the same time-point for 7 days, a CCK-8 assay was performed with a Cell Counting Kit-8 (Beyotime Institute of Biotechnology, Haimen, China). Briefly, the medium in each test well was replaced with 180 μl of fresh medium supplemented with 20 μl of CCK-8 reagent and incubated at 37 °C for 3 h in the dark. Then, the medium was transferred to a new 96-well plate, and the absorbance was measured at 450 nm with a microplate reader (EL×800; BioTek Instruments Inc., Highland Park, FL, USA).

### Alizarin red S staining

After induction for 14 days, the BMMSCs were washed with PBS and fixed in 4% paraformaldehyde for 30 min. Then, the cells were stained with Alizarin red S for 30 min. To remove excess staining solution, the cells were washed with PBS three times, and the stained samples were observed and photographed with an inverted microscope (Olympus, Shinjuku City, Tokyo, Japan). Quantitative analysis was conducted by dissolving the stained samples into 2% cetylpyridinium chloride and measuring the OD values of the solutions at 560 nm.

### Oil red O staining

After 7 days of adipogenic induction, the cells were fixed with 4% paraformaldehyde and stained with Oil red O for 30 min. The stained cells were observed and photographed under a microscope. For quantification, the stained samples were dissolved with isopropanol and the OD values of the solutions were measured at 560 nm.

### Alcian blue staining

After chondrogenic induction for 7 days, the cells were fixed with 4% paraformaldehyde and stained with Alcian blue for 20 min. Then, the stained cells were observed and photographed.

### Statistical analysis

All data were collected from at least three independent experiments (biological replicates) with three repeats (technique repeats). We combined the quantitative data from three biological replicates and analyzed the results with GraphPad Prism 5 software (San Diego, CA, USA). Statistical significance between groups was determined by one-way analysis of variance (ANOVA) and Tukey’s post-hoc test. Data are presented as the mean ± standard deviation (SD). Values of *p* < 0.05 were considered statistically significant.

## Results

### Identification of the Mφ phenotypes

**Figure 1:**
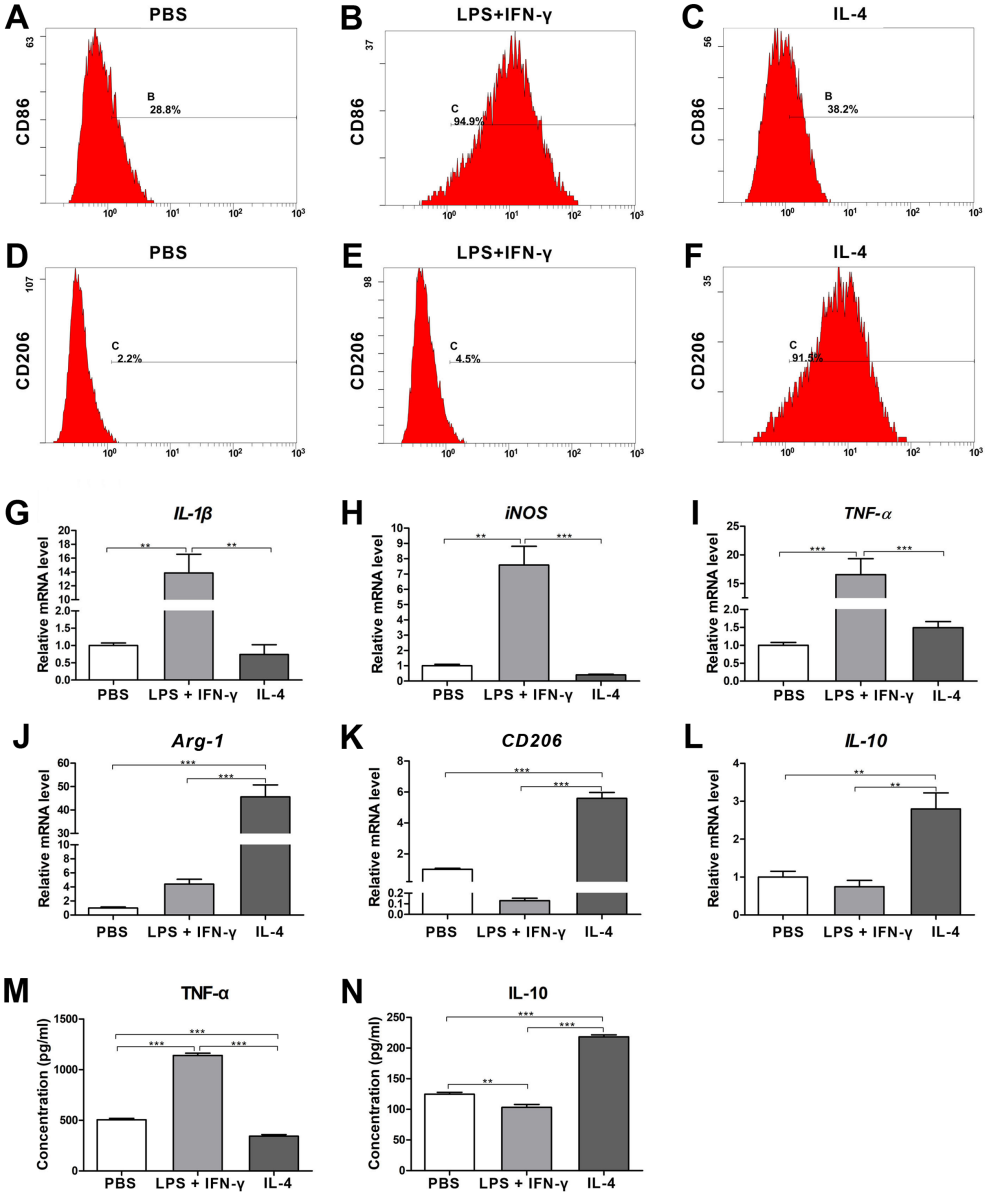
Identification of the macrophage phenotypes following stimulation with LPS plus IFN-γ (M1 induction) or IL-4 (M2 induction); unpolarized cells (incubated with medium supplemented with PBS) are considered M0 cells. (A–F) Results from the flow cytometry analysis of CD86 (M1 marker) and CD206 (M2 marker) in LPS plus IFN-γ-stimulated, IL-4-stimulated or unstimulated (PBS) cells. (G–L) Gene expression levels in LPS plus IFN-γ-stimulated, IL-4-stimulated or unstimulated (PBS) cells detected by qRT-PCR assay. *IL-*1β, *iNOS* and *TNF-*α were used as M1-related markers, while *Arg-1*, *CD206* and *IL-10* were applied as M2-related markers (values were normalized to β*-actin* and relative to PBS group (unstimulated cells)). (M and N) ELISA results of cytokine levels in the supernatants generated by LPS plus IFN-γ-stimulated, IL-4-stimulated or unstimulated (PBS) cells. TNF-α was used as an M1-polarization marker, and IL-10 was used as an M2-polarization marker. Data are presented as the mean ± SD for *n* = 3; **p < 0.01 and ***p < 0.001 indicate significant differences between the indicated columns.

After stimulation with different cytokines, the expression of the cell surface markers in Mφs with different phenotypes was analyzed by flow cytometry. Compared to PBS- and IL-4-treated Mφs, the cells treated with LPS plus IFN-γ showed significant upregulation of CD86 (specific surface marker of M1 Mφs, Figs. 1A–1C). Cells treated with IL-4 displayed higher expression of CD206 (specific surface marker of M2 Mφs) than did cells treated with PBS or LPS plus IFN-γ (Figs. 1D–1F). The mRNA expression levels of specific genes known as M1 and M2 markers (Murray & Wynn, 2011) were quantified by qRT-PCR. Compared with cells treated with PBS or IL-4, the cells treated with LPS plus IFN-γ had significantly upregulated expression levels of *IL-1*β, *iNOS* and *TNF-*α (M1-specific markers) (Figs. 1G–1I; *p* < 0.01 or 0.001). The levels of *Arg-1*, *CD206* and *IL-10* (M2-specific markers) in the cells treated with IL-4 were obviously higher than they were in the cells stimulated with PBS or LPS plus IFN-γ (Figs. 1J–1L; *p* < 0.01 or 0.001). In addition, the expression levels of *IL-1*β, *iNOS* and *TNF-*α in PBS- and IL-4-treated cells showed no significant difference, and there was no significant difference in the expression levels of *Arg-1*, *CD206* or *IL-10* between the cells treated with PBS and those treated with LPS plus IFN-γ (Figs. 1G–1L). The ELISA data revealed that the concentration of TNF-α in the CM generated by the LPS plus IFN-γ treated cells was significantly higher than it was in the CM treated by PBS and IL-4 (Fig. 1M; *p* < 0.001), a finding that was consistent with the results of PCR. Similarly, the concentration of IL-10 in the CM generated by IL-4-treated cells was significantly higher than it was in the CM for each of the other two groups (Fig. 1N; *p* < 0.001). All of these results suggested that the RAW264.7 cells were successfully induced to M1 polarization by LPS plus IFN-γ and to M2 polarization by IL-4.

### Characterization of different Mφ-derived exosomes

**Figure 2:**
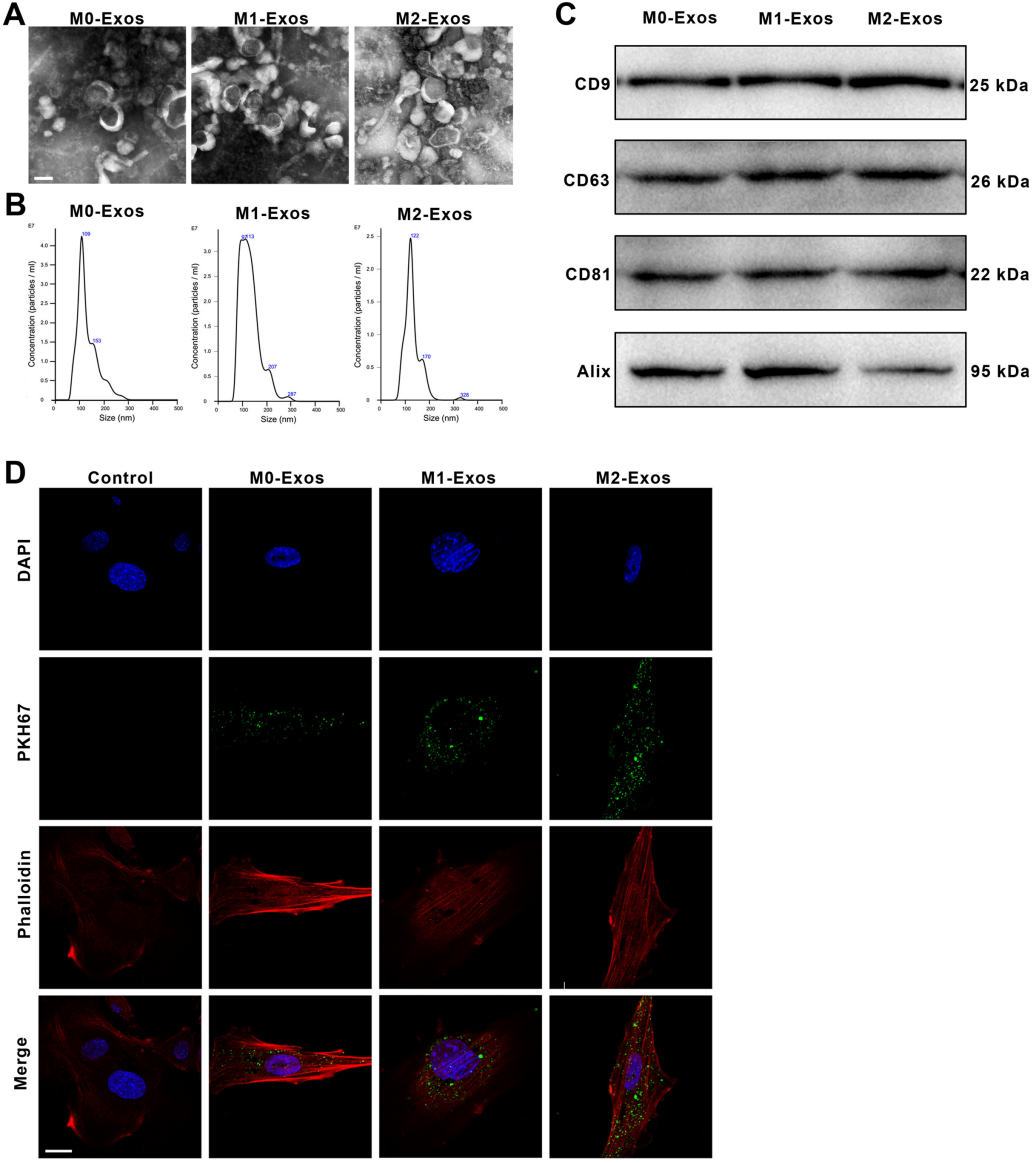
Identification of the exosomes derived from the polarized macrophages (M1-Exos and M2-Exos) or the unpolarized macrophages (M0-Exos). (A) Representative TEM images of the M0-Exos, M1-Exos and M2-Exos (scale bar: 100 nm). (B) ****Size distribution profiles of the M0-Exos, M1-Exos and M2-Exos, as determined by nanoparticle tracking analysis. (C) ****The presence of exosome marker proteins (CD81, CD63, CD9 and Alix) in the M0-Exos, M1-Exos and M2-Exos (Western blot assay results). (D) Representative confocal images showing exosomes endocytosed by the BMMSCs (the BMMSCs were incubated with PKH67-labeled M0-Exos, M1-Exos or M2-Exos for 4 h; the cells cultured without exosomes served as the blank control; scale bar: 10 μm): green, the PKH67-labeled exosomes; red (phalloidin), the framework of the BMMSCs. TEM, transmission electron microscopy.

The exosomes were isolated from the CM generated by M0, M1 or M2 Mφs. The images viewed by transmission electron microscopy showed that exosomes released from Mφs were small round nanometer-sized particles with bilayer membranes (Fig. 2A). Nanoparticle tracking analysis revealed that the diameters of these exosomes ranged from 30 to 150 nm (Fig. 2B). Moreover, the M0-Exos, M1-Exos and M2-Exos all expressed the exosomal markers CD9, CD63, CD81 and Alix (Fig. 2C). These all confirmed the successful extraction of exosomes.

### Internalization of the exosomes by BMMSCs

To visualize whether M0-Exos, M1-Exos and M2-Exos can be internalized by BMMSCs, PKH67-labeled exosomes were incubated with BMMSCs. The endocytosis of the exosomes was observed under a confocal microscope. The green fluorescence of the PKH67-labeled exosomes could be observed in the cells cocultured with exosomes, while the cells not cocultured with exosomes presented only the blue DAPI fluorescence and the red phalloidin fluorescence (Fig. 2D). These demonstrated that M0-Exos, M1-Exos and M2-Exos all could be untaken and internalized by BMMSCs.

### Effects of Mφ-derived exosomes on the proliferation of the BMMSCs

**Figure 3:**
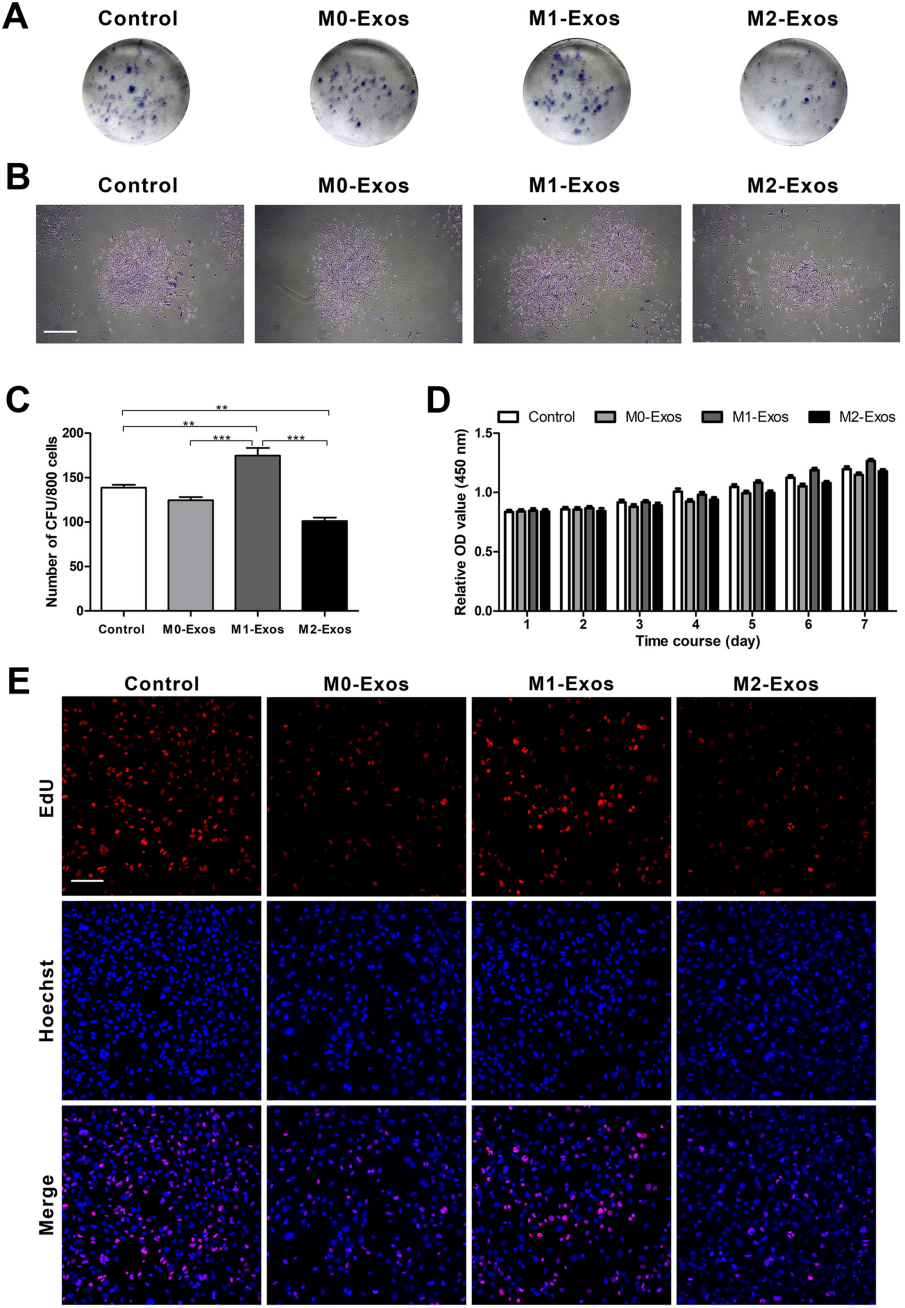
Cell proliferation in response to exosome-based incubation (control: cells in normal culture); BMMSCs were incubated in normal medium supplemented with exosomes (M0-Exos, M1-Exos or M2-Exos). (A) Representative general images of the colony formed by the BMMSCs after 14 days in various cultures. (B) A single colony formed by the BMMSCs (scale bar: 250 μm). (C) Results from the quantitative analysis of CFUs. (D) Proliferative potential of the BMMSCs in different cultures (during a 7-day incubation period) in terms of the CCK-8 assay results. (E) Proliferative potential of the BMMSCs in different cultures in terms of the EdU incorporation assay results; representative images showing EdU-positive cells (labeled with red fluorescence; scale bar: 100 μm). Data are presented as the mean ± SD for *n* = 3; **p < 0.01 and ***p < 0.001 indicate significant differences between the indicated columns.

To detect the proliferation ability of BMMSCs in different culture conditions, colony-forming assay was conducted firstly. Toluidine blue staining revealed that all of the groups had the ability to form new colony units (Figs. 3A and 3B). The data from the quantified analysis of CFU numbers showed that the supplement of M1-Exos promoted the BMMSCs to form the most CFUs (*p* < 0.01 or 0.001). However, M2-Exos obviously decreased the number of CFUs formed by the BMMSCs as compared with that of control (*p* < 0.01). The supplementation of M0-Exos did not have a significant influence on CFU formation of the BMMSCs (Fig. 3C). The data from CCK-8 assays conducted during the 7-day time course indicated no significant difference between different groups. However, the trend of the cell growth histograms was consistent with that of the colony-forming assays (Fig. 3D). The effects of exosomes on BMMSC proliferation were further confirmed by EdU incorporation assays. It is obvious that cells cultured with M1-Exos have the most EdU-positive cells, suggesting that cells in this group have the greatest proliferative potential. While cells cultured with M0-Exos and M2-Exos showed fewer EdU-positive cells than did the control group, revealing low proliferative potential (Fig. 3E).

### Effects of Mφ-derived exosomes on the osteogenic differentiation of the BMMSCs

**Figure 4:**
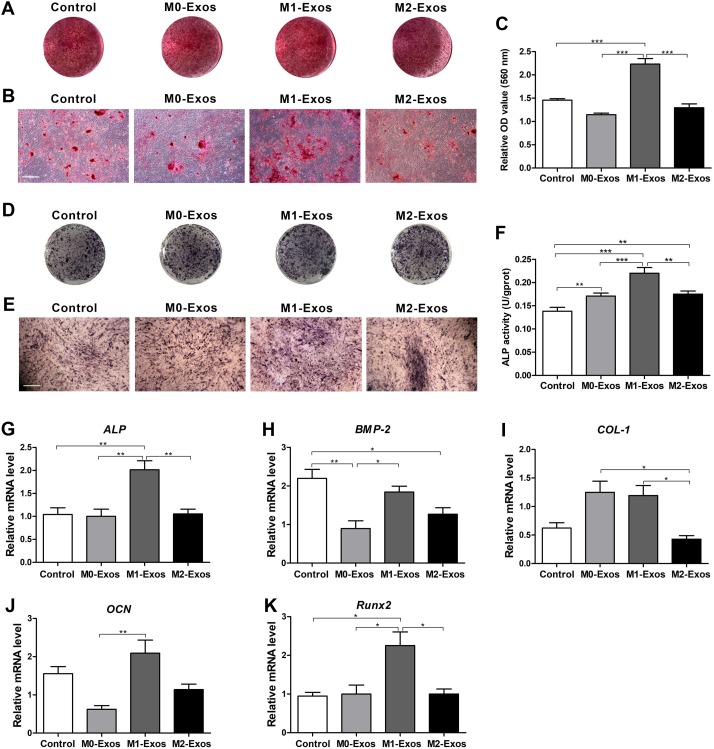
Osteogenic potential of BMMSCs in response to exosome-based incubation (control: cells in normal osteogenic cultures); BMMSCs were incubated in normal osteogenic medium supplemented with exosomes (M0-Exos, M1-Exos or M2-Exos). (A) General view of Alizarin red S stained cells after 14 days of osteogenic induction. (B) Representative images of Alizarin red S staining captured under the microscope (scale bar: 250 μm). (C) Quantitative analysis results of mineralized nodules. (D) General view of ALP staining after 7 days of osteogenic induction. (E) Representative images of ALP staining captured under the microscope (scale bar: 250 μm). (F) Statistical analysis results of the ALP activity. (G–K) Expression levels of osteogenesis-related genes (*ALP*, *BMP-2*, *COL-1, OCN* and *Runx2*) in the BMMSCs (qRT-PCR assay). Values were normalized to the level of β*-Actin*. Data are presented as the mean ± SD; *n* = 3; *p < 0.05, **p < 0.01 and ***p < 0.001 indicate significant differences between the indicated columns.

To analyze the osteogenic differentiation ability of the BMMSCs under different culture conditions, Alizarin red S staining was conducted on day 14. It can be found that BMMSCs cultured with supplement of M1-Exos appeared to form the most calcium deposits. However, the cells cultured with supplement of M0-Exos and M2-Exos formed fewer calcium deposits than that of the control (Figs. 4A and 4B). The results of quantitative analysis also indicated that M1-Exos significantly promoted the mineralized nodule formation of BMMSCs (*p* < 0.001), while M0-Exos and M2-Exos showed no significant influence on the mineralized nodule formation of BMMSCs as compared with the control group (Fig. 4C). These were further demonstrated by ALP staining (Figs. 4D and 4E) and the detection of ALP activity on day 7. The quantitative analysis of ALP activity revealed that M0-Exos, M1-Exos and M2-Exos all increased the ALP activity of BMMSCs (Fig. 4F; *p* < 0.01 or 0.001), and it can be found that cells cultured with supplement of M1-Exos exhibited the highest ALP activity (*p* < 0.001). The mRNA expression levels of osteogenesis-related genes were analyzed by qRT-PCR after cells were subjected to osteogenic induction for 7 days. The data showed that neither M0-Exos or M2-Exos significantly influenced the expression levels of *ALP, COL-1, OCN* and *Runx2* but reduced the expression level of *BMP-2* (*p* < 0.05 or 0.01). While the expression levels of both *ALP* and *Runx2* in the cells cultured with M1-Exos were increased (*p* < 0.05 or 0.01). In addition, the expression levels of *ALP, BMP-2, OCN* and *Runx2* in the cells cultured with M1-Exos were higher than those of cells cultured in M0-Exos or M2-Exos (Figs. 4G–4K; *p* < 0.05 or 0.01).

### Effects of Mφ-derived exosomes on the adipogenic differentiation of the BMMSCs

**Figure 5:**
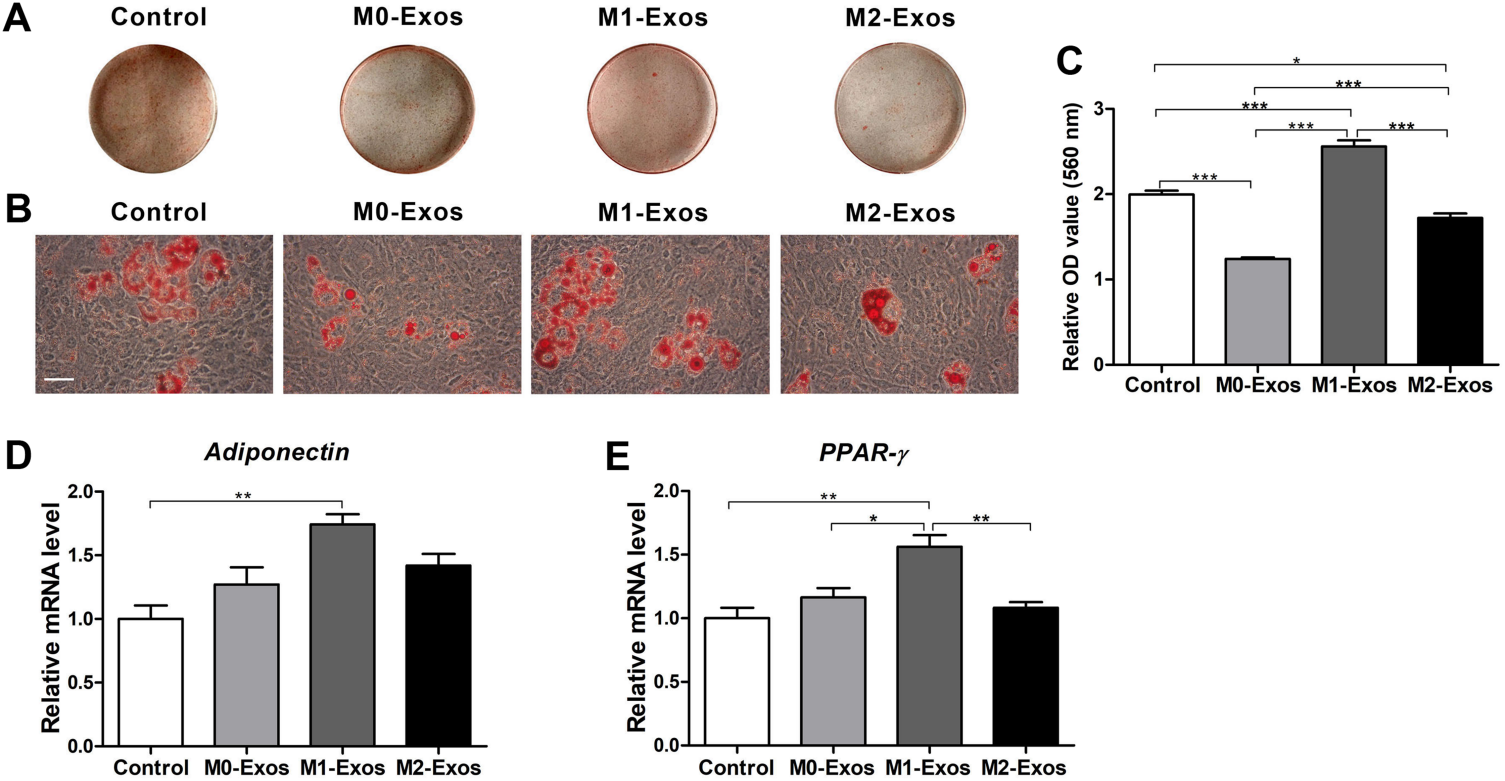
Adipogenic potential of the BMMSCs in response to exosome-based incubation (control: cells in normal adipogenic cultures); BMMSCs were incubated in normal adipogenic medium supplemented with exosomes (M0-Exos, M1-Exos or M2-Exos). (A) General view of the Oil red O-stained lipid droplets after 7 days of adipogenic induction. (B) Representative images of Oil red O staining captured under the microscope (scale bar: 100 μm). (C) ****Quantitative analysis results of the lipid droplets. (D and E) Relative mRNA expression levels of adipogenesis-related genes (*adiponectin* and *PPAR-*γ) in the BMMSCs (qRT-PCR assay results). Values were normalized to β-*Actin* and relative to the level of the control. Data are presented as the mean ± SD; *n* = 3; *p < 0.05, **p < 0.01 and ***p < 0.001 indicate significant differences between the indicated columns.

To investigate the adipogenic differentiation ability of BMMSCs under different culture conditions, after induction for 7 days, the cells were stained with Oil red O. The results revealed that the cells treated with M1-Exos formed the most lipid droplets, while both the M0-Exo- and M2-Exo-incubated cells formed fewer lipid droplets than were found in the control group (Figs. 5A and 5B). The quantitative analysis data were consistent with the staining results. Both M0-Exos and M2-Exos had negative effects on the lipid droplet formation of the BMMSCs (*p* < 0.001 or 0.05). While M1-Exos significantly promoted more lipid droplets to form in the BMMSCs (Fig. 5C; *p* < 0.001). The effects of exosomes on the adipogenic differentiation of BMMSCs were further confirmed by qRT-PCR analysis. It can be found that M1-Exos dramatically upregulated the *adiponectin* and *PPAR-*γ gene (adipogenesis-related markers) expression of BMMSCs (*p* < 0.01), while cells cultured with M0-Exos and M2-Exos showed no difference in *adiponectin* and *PPAR-*γ gene expression compared with that of the control cells. Moreover, the *PPAR-*γ expression level in the cells cultured with M1-Exos was also higher than that of the cells cultured with M0-Exos or M2-Exos (Figs. 5D and 5E; *p* < 0.05 or 0.01).

### Effects of the Mφ-derived exosomes on the chondrogenic differentiation of the BMMSCs

**Figure 6:**
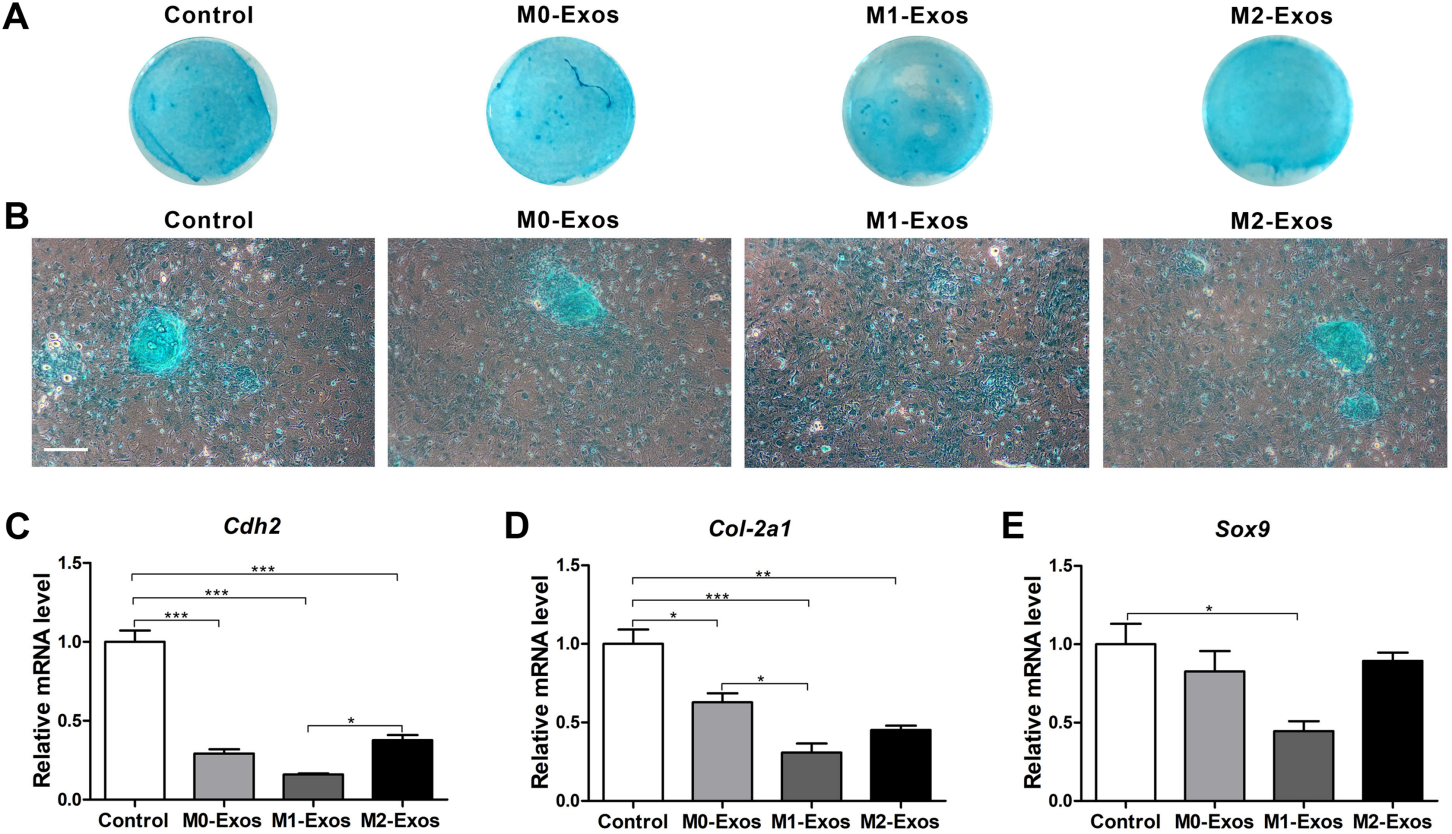
Chondrogenic potential of the BMMSCs in response to exosome-based incubation (control: cells in normal chondrogenic cultures); BMMSCs were incubated in normal chondrogenic medium supplemented with exosomes (M0-Exos, M1-Exos or M2-Exos). (A) General view of Alcian blue staining after 7 days of chondrogenic induction. (B) Representative images of Alcian blue staining captured under the microscope (scale bar: 250 μm). (C–E) Expression levels of chondrogenesis-related genes (*Cdh2*, *Col-2a1* and *Sox9*) in the BMMSCs after 7 days of chondrogenic induction (qRT-PCR assay). Data are presented as the mean ± SD; *n* = 3; *p < 0.05, **p < 0.01 and ***p < 0.001 indicate significant differences between the indicated columns.

The chondrogenesis ability of the BMMSCs in different cultures was detected by Alcian blue staining. It is obvious that cells cultured in normal culture medium formed more significant and larger multilayered aggregates than did the other groups of cells. However, the cells cultured in M1-Exos only formed small monolayered aggregates (Figs. 6A and 6B). The expression level of chondrogenesis-related genes *Cdh2* (cadherin 2), *Col2-a1* (collagen II-encoding gene) and *Sox9* (SRY (sex-determining region Y)-box 9) were also analyzed by qRT-PCR. It was discovered that M0-Exos, M1-Exos and M2-Exos downregulated the expression of chondrogenesis-related genes. Concretely, both M0-Exos and M2-Exos had a negative effect on the expression levels of *Cdh2* and *Col2-a1* (*p* < 0.001, 0.01 or 0.05) but showed no significant influence on *Sox9* expression. M1-Exos significantly downregulated the expression level of all the detected chondrogenesis-related genes compared with the level expressed in the control group cells (Figs. 6C–6E; *p* < 0.001 or 0.05). These results indicate that Mφ-derived exosomes have a negative effect on BMMSC chondrogenic differentiation.

## Discussion

Mφs are key mediators of host defense and participate in a range of physiological process (Oishi & Manabe, 2018). They are heterogenous cells which can influence the microenvironment through their polarization into different phenotypes (Brown, Sicari & Badylak, 2014). Over the last few years, the importance of Mφs in stem cell survival and tissue repair has been recognized (Cai et al., 2018). Since Mφs can switch their phenotypes during the process of tissue regeneration (Novak & Koh, 2013), it’s essential to explore the regulating effect of different phenotypes of Mφs on MSCs. In this study, RAW264.7 cells were stimulated with different cytokines and different methods were used to identified their phenotypes. The results of flow cytometry analysis, ELISA and qRT-PCR all revealed that RAW264.7 cells were successfully polarized to M1phenotype with the stimulation of LPS plus IFN-γ or polarized to M2 phenotype with the stimulation of IL-4. This was consistent with the results of our previous study (He et al., 2018).

Recently, several studies have confirmed the regulation effect of macrophage-derived exosomes. For example, it was reported that Mφ-derived extracellular vesicles (EVs) are essential for intestinal stem cell self-renewal, proliferation and intestinal homoeostasis (Saha et al., 2016). Another study established that Mφ-derived exosomes can accelerate wound repair by inducing endothelial cell proliferation and migration (Li et al., 2019). However, it still remains unclear whether Mφ-derived exosomes also play a role in the regulation of BMMSC property. To test this assumption, exosomes were isolated from the CM of M0, M1 or M2 Mφs, separately. The results of TEM and NTA showed that the size and morphology of these isolated exosomes meet the standards mentioned in the literature (Kalluri & LeBleu, 2020) and the exosmal markers are also positive in these exosomes. This demonstrated that exosomes were successfully isolated from M0, M1 and M2 Mφs. In addition, it was found that the exosomes secreted by different phenotypes of Mφs all could be internalized by the BMMSCs.

Based on these results, the effects of exosomes derived from M0, M1 and M2 Mφs on BMMSC proliferation and osteogenic, adipogenic and chondrogenic differentiation were investigated. Our data demonstrated that the exosomes secreted by M1 Mφs promoted the proliferation of BMMSCs, however, the exosomes secreted by M2 Mφs impaired the proliferation of BMMSCs. M0-Exos didn’t exhibit significant influence on the proliferation of BMMSCs (Figs. 3A–3C). This observation is consistent with our previous findings (He et al., 2018) and indicates that exosomes are key mediators during the regulating of Mφ-derived CM on BMMSC proliferation. However, it’s in contrast to previously reported data that IFN-γ-activated Mφs negatively regulated the proliferation and activation of hematopoietic stem cells (McCabe et al., 2015), while M2 Mφs positively regulated the proliferation of BMMSCs (Yu et al., 2016). This discrepancy can be attributed to the differences in cell lineage and culture conditions.

Thus far, the exact role of differently polarized Mφs in osteogenesis has not reached a consensus (Pajarinen et al., 2019). It has been demonstrated that CM of classically activated monocytes can increase the expression of osteogenic genes in human MSCs (Omar et al., 2011). Zhang et al. (2017b) also found that M1 Mφs can promote the osteogenic of the MSCs during the early and middle stages. In this study, we confirmed that Mφ-derived exosomes also play key roles in the osteogenic differentiation of BMMSCs. The data of Alizarin red S staining, ALP activity assays and gene expression measures all demonstrated that M1-Exos can significantly promote the osteogenic differentiation of BMMSCs (Fig. 4). This supported the results of the aforementioned studies. However, we found that neither M0-Exos nor M2-Exos exhibited an obvious effect on the osteogenesis differentiation of the BMMSCs according to the Alizarin red S staining or gene expression evidence. It was inconsistent with our previous study which proved that CM generated by M0 or M2 Mφs can positively regulate the osteogenesis of BMMSCs (He et al., 2018). In fact, several papers have reported that M2 Mφs can promote osteogenic differentiation of MSCs (Schlundt et al., 2015; Jin et al., 2019; Zhu et al., 2019). In this study, only the results of the ALP activity assay showed a slight promotion effect by M0-Exos and M2-Exos on BMMSC osteogenic differentiation. This revealed that the exosomes and CM derived from the same phenotype of Mφs didn’t exert the same influence on the osteogenic differentiation of BMMSCs. It’s a reminder that other mediators in the CM of Mφs may also affect the property of BMMSCs and Mφs with different phenotypes may modulate the osteogenesis of BMMSCs via different paracrine components. M1 Mφs may promote the early and middle stages of osteogenesis mainly through exosomes. M0 and M2 Mφs may regulate the osteogenic differentiation of BMMSCs by secreting cytokines during the late stage.

Mφs also play crucial roles in adipose tissue and influence the adipogenesis. It was reported that M1 Mφs suppressed the adipogenesis of PDGFRα+ preadipocytes, but M2 Mφs showed no influence (Cheng et al., 2019). In addition, researchers discovered that Mφs in adipose tissue can transport miRNAs through exosomes which finally influence the insulin sensitivity and glucose homeostasis (Ying et al., 2017). Evidence also confirmed that Mφ-derived microRNA can influence adipocyte metabolism (Tryggestad et al., 2019). These all indicate that Mφ-derived exosomes may also participate in the adipogenic differentiation of MSCs. This hypothesis was confirmed in the present study. We discovered that M1-Exos promoted the adipogenic differentiation of the BMMSCs, according to Oil red O staining and gene expression levels. However, M0-Exos and M2-Exos did not have a significant influence on adipogenesis-related gene expression and even reduced the lipid droplet formation in the BMMSCs. The result of M1-Exos was in contradiction with that of Cheng et al. (2019), this could be due to the difference of cocultured cells and the involvement of other mediators in the co-culture condition of Cheng et al. (2019). Nevertheless, it supports the data of our previous study which showed that the CM of M1 Mφs promoted the adipogenic differentiation of BMMSCs (He et al., 2018). This demonstrated that exosomes also modulate the effect of Mφ-derived CM on the adipogenic differentiation of BMMSCs.

Apart from proliferation and osteogenic and adipogenic differentiation, Mφs are also involved in the chondrogenic differentiation of stem cells. Several studies have confirmed the negative effect of M1 Mφs on chondrogenesis and the chondrogenesis-inductive effect of M2 Mφs (Han et al., 2014; Dai et al., 2018; Hu et al., 2018). However, the exact mechanism for these effects remains unclear. Sesia et al. (2015) found that Mφs only promoted the chondrogenic differentiation of BMMSCs in the condition of direct coculture and the CM of Mφs did not modulate chondrogenesis. However, others reported that the CM generated by M1 Mφs inhibited the chondrogenesis of MSCs and that the CM of M2 Mφs did not influence the expression of the *COL2* gene but reduced the expression of the *ACAN* gene (Fahy et al., 2014). In this study, the effects of exosomes derived from different phenotypes of Mφs on the chondrogenic differentiation of BMMSCs were also investigated. The results of cell staining and the detect of chondrogenesis-related genes both demonstrated that exosomes derived from M0, M1 and M2 Mφs all had a negative effect on BMMSC chondrogenesis. The effect of M1-Exos was consistent with the results of Fahy et al. (2014), but the beneficial effect of M2-Exos was not found in our study. On one hand, it may be attributed to the different origin of Mφs, and on the other hand, it’s probably because of the involvement of other cytokines in the CM. However, our study detected chondrogenic differentiation of BMMSCs only at the early stage (on the 7th day). The long-term effect of Mφ-derived exosomes on chondrogenesis remains to be explored.

Taken together, the aforementioned data demonstrated that exosomes derived from different phenotypes of Mφs could exert various influences on the proliferation and osteogenic, adipogenic and chondrogenic differentiation of BMMSCs. It was found that M1-Exos exhibited more robust effects on the proliferation and osteogenic and adipogenic differentiation of BMMSCs than does M0-Exos or M2-Exos. In addition, all three types of exosomes had a suppressive effect on the chondrogenic differentiation of BMMSCs. This indicates that Mφ-derived exosomes may be explored as active reagents to improve the property of MSCs in the regenerative microenvironment.

## Conclusions

This study confirmed our hypothesis that exosomes modulate the effect of Mφ-derived CM on the proliferation and differentiation of BMMSCs. It was also found that even when derived from the same Mφ phenotype, the CM and exosomes do not necessarily exert similar cellular influences on the cocultured stem cells. This provides new insight into the interaction between Mφs and MSCs and indicates that Mφ-derived exosomes may be used in an efficient therapeutic strategy for tissue regeneration. However, the process of tissue repair and regeneration is successive, and it is difficult to determine the exact dose and timing for exosome application. In addition, the exact mechanisms of the exosomes still need to be further investigated.

## Supplemental Information

10.7717/peerj.8970/supp-1 Supplemental Information 1 Characterization of the BMMSCs isolated from the bone marrow of C57BL/6 mice. (A) Results from the flow cytometry analysis of cell surface markers of the BMMSCs. (B) Colony formation ability of the BMMSCs: a general view of colonies (top) and a single colony observed by microscopy (bottom; scale bar: 250 μm). (C) Representative images of EdU-positive cells (cell viability in terms of the EdU assay results; scale bar: 100 μm). (D) Growth curve of the BMMSCs during 7-days in culture in terms of the CCK-8 assay results. (E) A representative image showing the potential of the BMMSCs toward osteogenic differentiation (Alizarin red staining; scale bar: 250 μm). (F) A representative image showing the potential of the BMMSCs toward adipogenic differentiation (Oil red O staining; scale bar: 100 μm). (G) A representative image showing the potential of the BMMSCs toward chondrogenic differentiation (Alcian blue staining; scale bar: 100 μm).

10.7717/peerj.8970/supp-2 Supplemental Information 2 Raw data for Figures 1B, 1C, 3B, 3C, 4B 4D-I, 5B-D, 6B-D.

10.7717/peerj.8970/supp-3 Supplemental Information 3 Images of western blots.

## Additional Information and Declarations

### Data Availability

The following information was supplied regarding data availability: The raw data are available in the Supplemental Files.
