# Supplementary figures and images for: Exosomes derived from M0, M1 and M2 macrophages exert distinct influences on the proliferation and differentiation of mesenchymal stem cells
Source: PeerJ. 2020 Apr 24;8:e8970. doi: 10.7717/peerj.8970 (PMC7185029; doi:10.7717/peerj.8970)

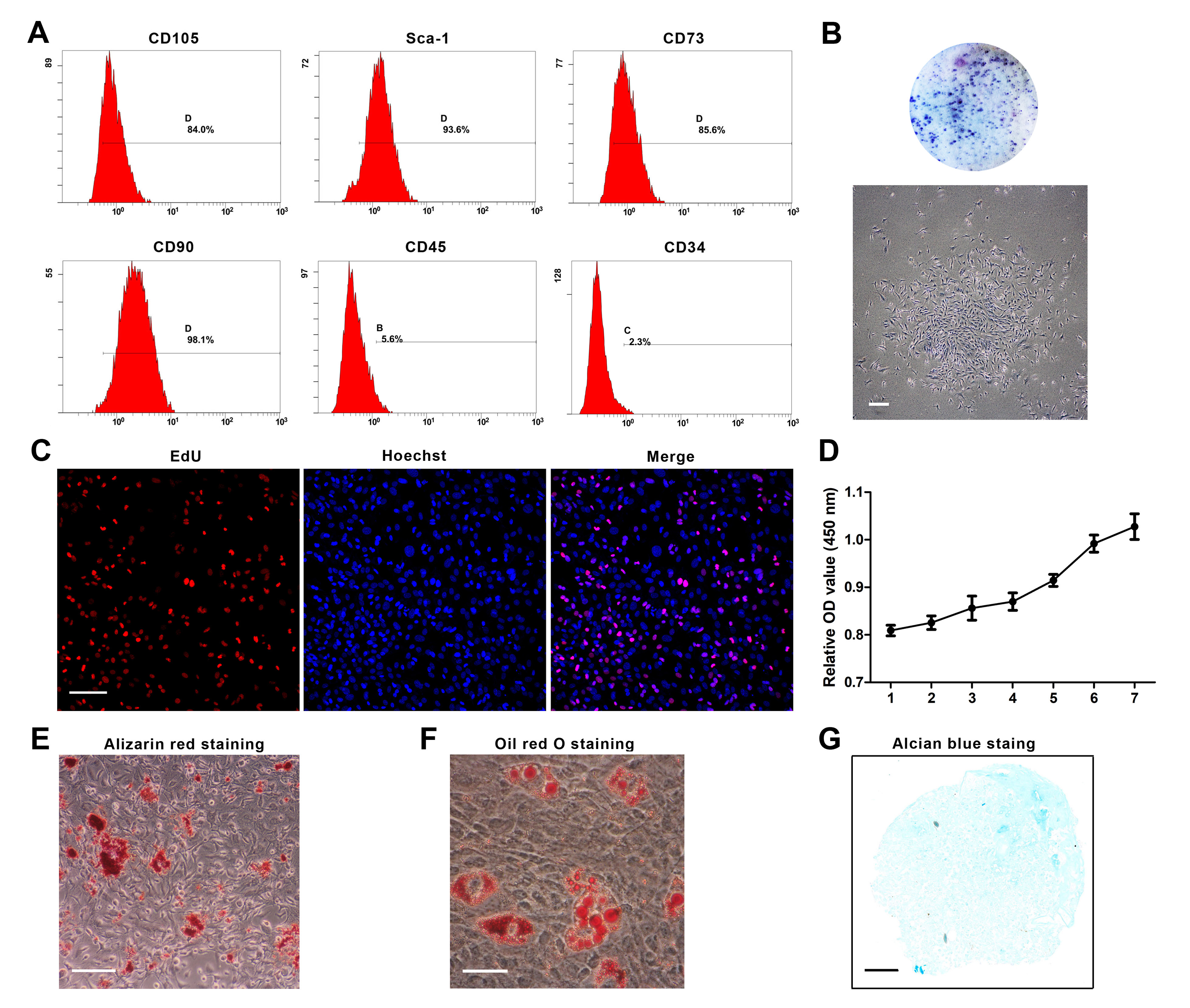

Supplement: Supplemental Information 1 — (A) Results from the flow cytometry analysis of cell surface markers of the BMMSCs. (B) Colony formation ability of the BMMSCs: a general view of colonies (top) and a single colony observed by microscopy (bottom; scale bar: 250 μm). (C) Representative images of EdU-positive cells (cell viability in terms of the EdU assay results; scale bar: 100 μm). (D) Growth curve of the BMMSCs during 7-days in culture in terms of the CCK-8 assay results. (E) A representative image showing the potential of the BMMSCs toward osteogenic differentiation (Alizarin red staining; scale bar: 250 μm). (F) A representative image showing the potential of the BMMSCs toward adipogenic differentiation (Oil red O staining; scale bar: 100 μm). (G) A representative image showing the potential of the BMMSCs toward chondrogenic differentiation (Alcian blue staining; scale bar: 100 μm). [file peerj-08-8970-s001.png]

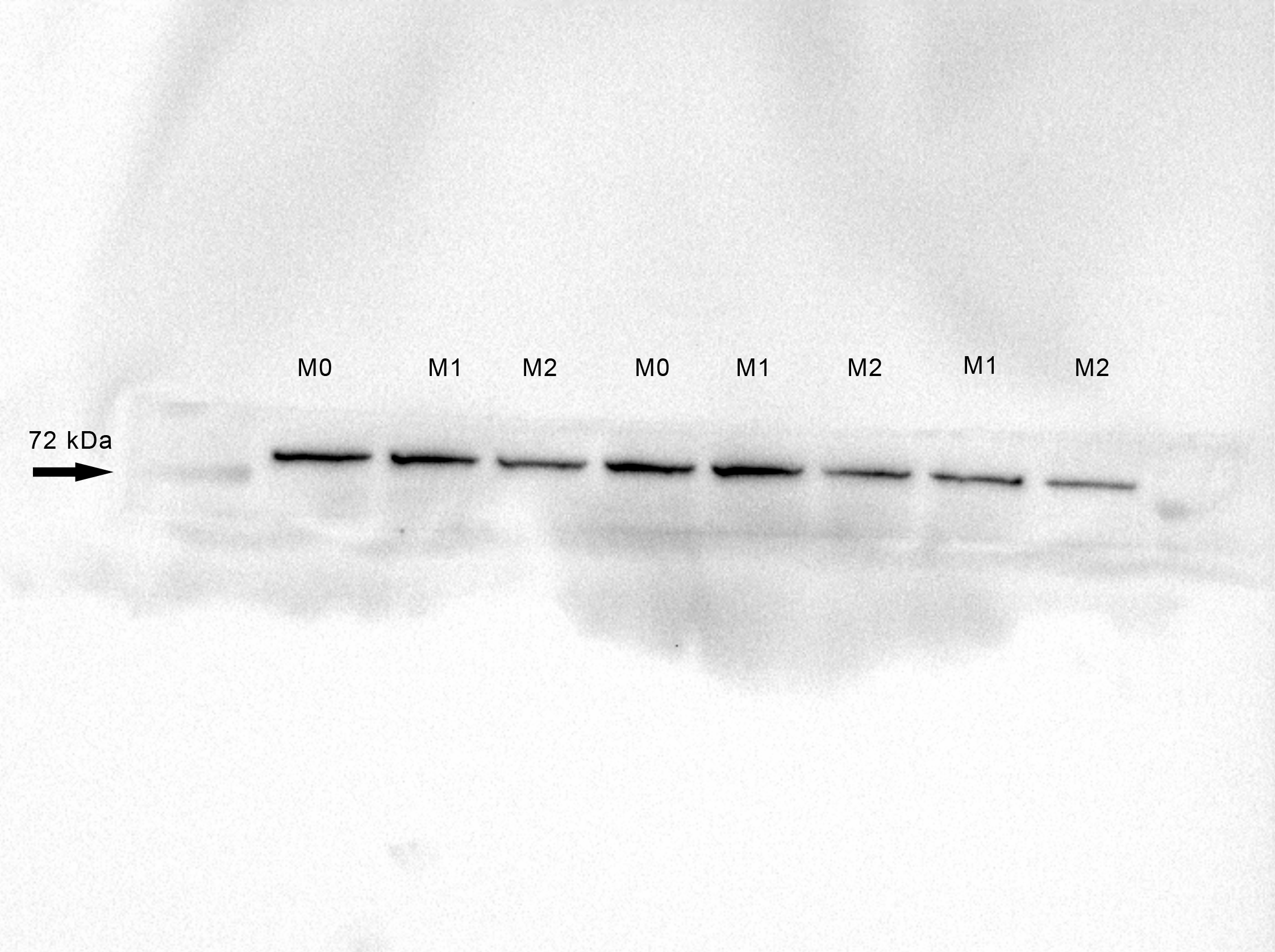

Supplement: Supplemental Information 3 [file peerj-08-8970-s003.zip › Western Blot/Alix.tif]

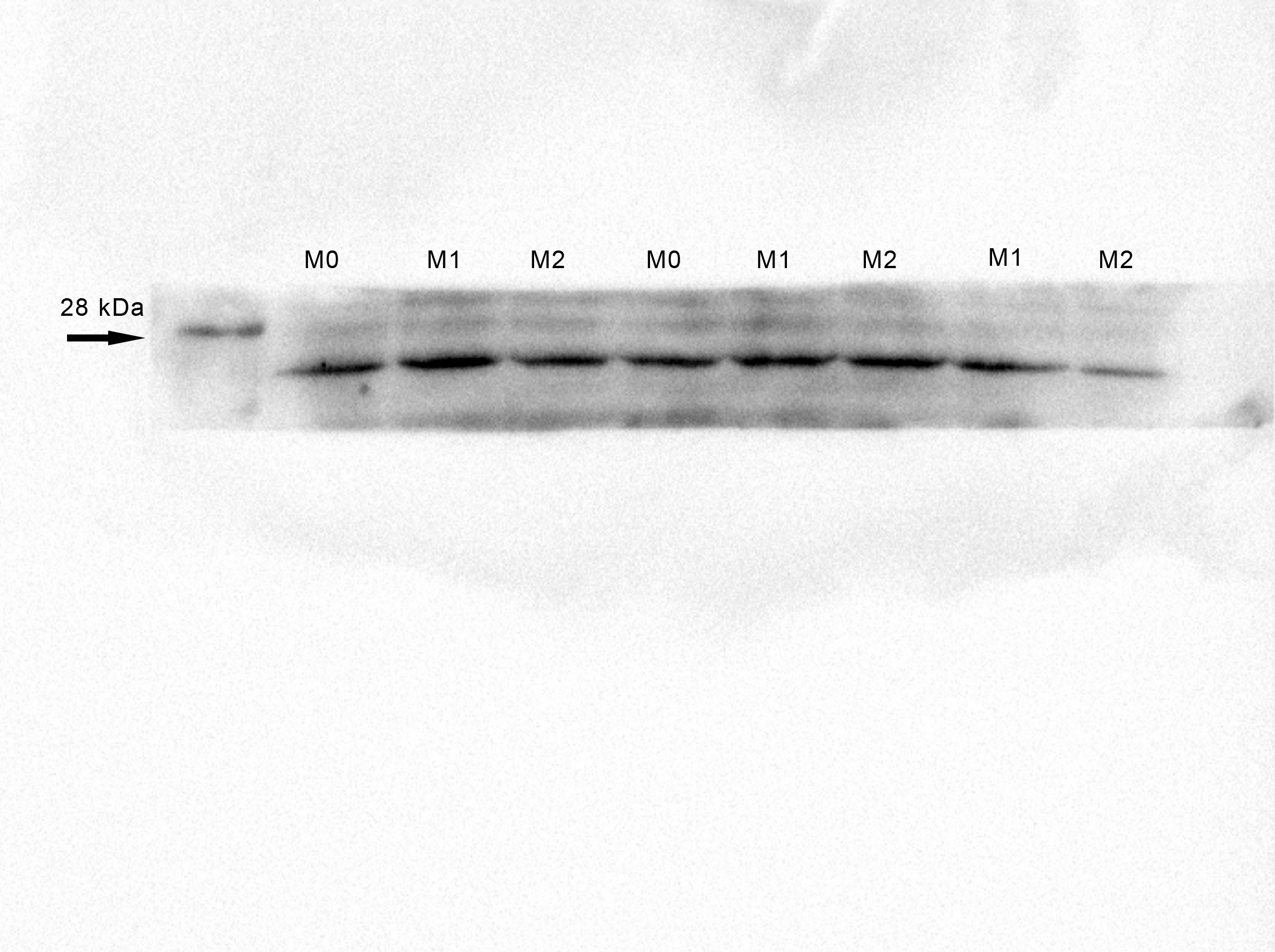

Supplement: Supplemental Information 3 [file peerj-08-8970-s003.zip › Western Blot/CD63.tif]

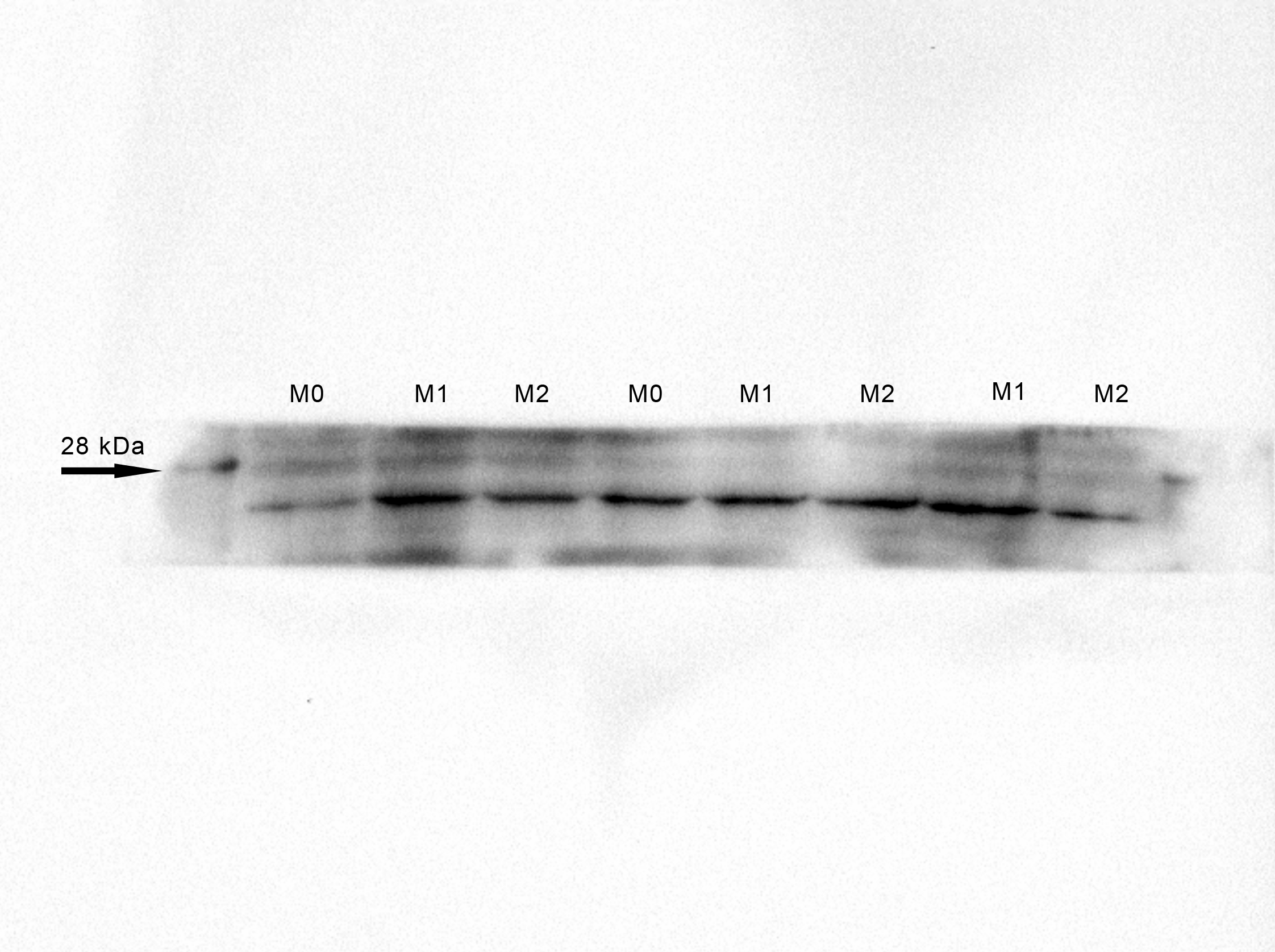

Supplement: Supplemental Information 3 [file peerj-08-8970-s003.zip › Western Blot/CD81.tif]

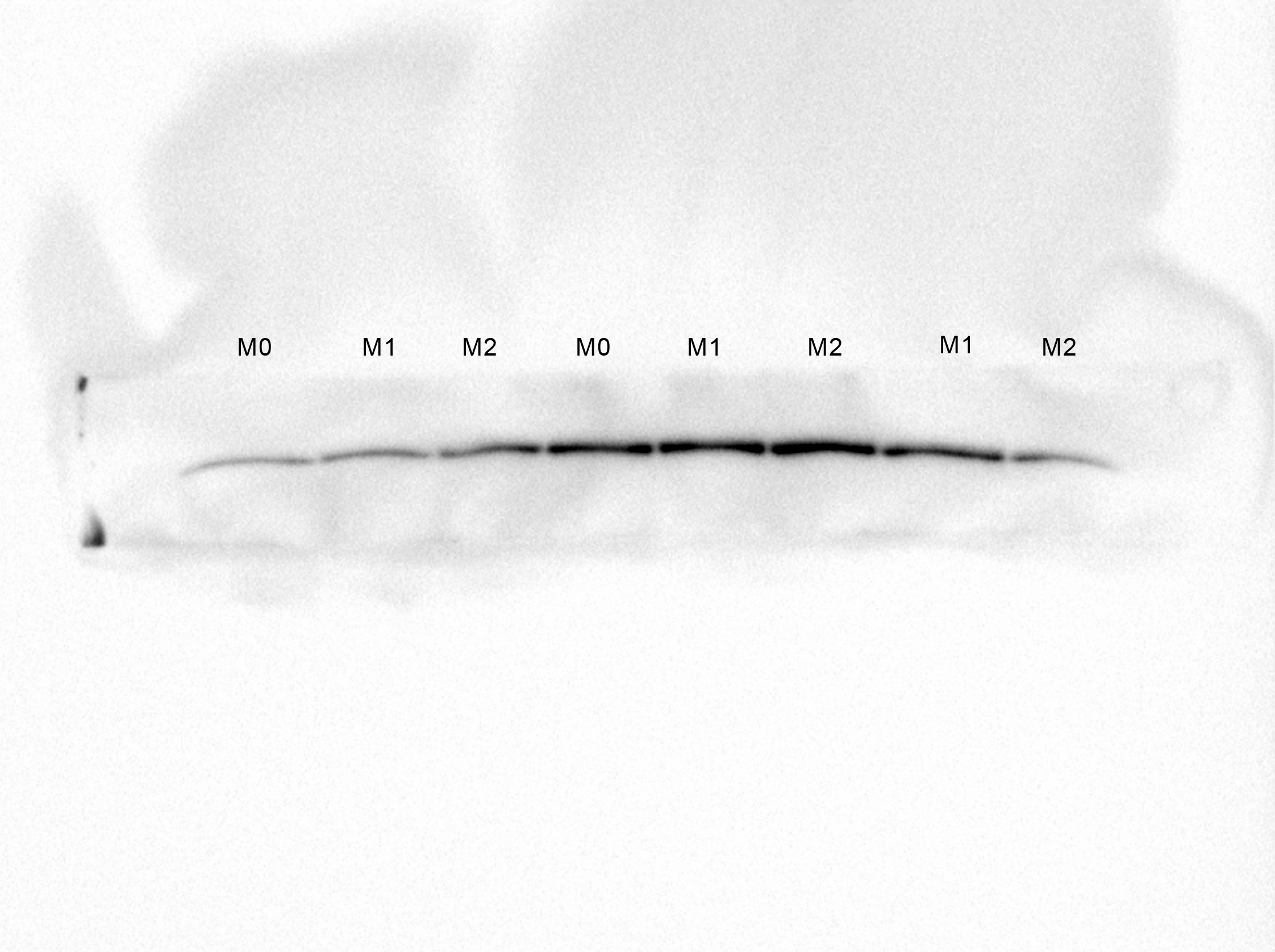

Supplement: Supplemental Information 3 [file peerj-08-8970-s003.zip › Western Blot/CD9.tif]
